# Influence of end‐tidal CO_2_ on cerebral blood flow during orthostatic stress in controls and adults with myalgic encephalomyelitis/chronic fatigue syndrome

**DOI:** 10.14814/phy2.15639

**Published:** 2023-09-09

**Authors:** C. (Linda) M. C. van Campen, Peter C. Rowe, Freek W. A. Verheugt, Frans C. Visser

**Affiliations:** ^1^ Stichting CardioZorg Hoofddorp The Netherlands; ^2^ Department of Paediatrics Johns Hopkins University School of Medicine Baltimore Maryland USA; ^3^ Onze Lieve Vrouwe Gasthuis (OLVG) Amsterdam The Netherlands

**Keywords:** carbon dioxide, myalgic encephalomyelitis/chronic fatigue syndrome, orthostatic intolerance, postural hyperventilation, tilt table test

## Abstract

Brain perfusion is sensitive to changes in CO_2_ levels (CO_2_ reactivity). Previously, we showed a pathological cerebral blood flow (CBF) reduction in the majority of myalgic encephalomyelitis/chronic fatigue syndrome (ME/CFS) patients during orthostatic stress. Limited data are available on the relation between CO_2_ and CBF changes in ME/CFS patients. Therefore, we studied this relation between ME/CFS patients and healthy controls (HC) during tilt testing. In this retrospective study, supine and end‐tilt CBF, as measured by extracranial Doppler flow, were compared with P_ET_CO_2_ data in female patients either with a normal heart rate and blood pressure (HR/BP) response or with postural orthostatic tachycardia syndrome (POTS), and in HC. Five hundred thirty‐five female ME/CFS patients and 34 HC were included. Both in supine position and at end‐tilt, there was a significant relation between CBF and P_ET_CO_2_ in patients (*p* < 0.0001), without differences between patients with a normal HR/BP response and with POTS. The relations between the %CBF change and the P_ET_CO_2_ reduction were both significant in patients and HC (*p* < 0.0001 and *p* = 0.0012, respectively). In a multiple regression analysis, the patient/HC status and P_ET_CO_2_ predicted CBF. The contribution of the P_ET_CO_2_ to CBF changes was limited, with low adjusted *R* ^2^ values. In female ME/CFS patients, CO2 reactivity, as measured during orthostatic stress testing, is similar to that of HC and is independent of the type of hemodynamic abnormality. However, the influence of CO_2_ changes on CBF changes is modest in female ME/CFS patients.

## Graphical abstract

We studied the relation between cerebral blood flow and end‐tidal CO2 changes by orthostatic stress testing and compared the patients (n=535) with 43 female controls. The influence of factors such as disease duration as well as disease severity were studied in a multiple regression analysis.

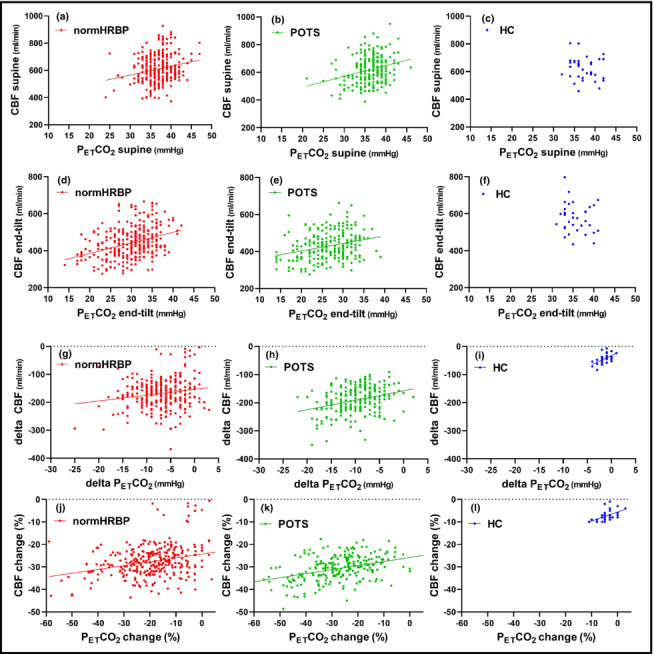

## 1 INTRODUCTION

Cerebral vascular CO_2_ reactivity denotes the ability of cerebral vessels to dilate or constrict in response to changes in *p*CO_2_. In the 1940s, Kety and Schmidt demonstrated a decrease in cerebral blood flow (CBF) during hypocapnia and an increase in cerebral blood flow (CBF) during hypercapnia (Kety & Schmidt, 1946, 1948). Since then, multiple studies using a variety of techniques (Juttukonda & Donahue, 2019) have measured this cerebrovascular CO_2_ reactivity in a variety of diseases and healthy controls (Liu et al., 2019).

We have previously demonstrated in myalgic encephalomyelitis/chronic fatigue syndrome (ME/CFS) patients that CBF is significantly decreased during a 70‐degree tilt test compared with healthy controls (van Campen, Verheugt, et al., 2020). The abnormal CBF reduction was also observed in these patients during a mild orthostatic stress of a 20‐degree tilt test and during sitting (van Campen, Rowe, & Visser, 2020a, 2020b). The pathophysiology of the abnormal CBF reduction is not yet fully elucidated but may involve an abnormal cardiac output reduction due to venous pooling (Del Pozzi et al., 2014; van Campen & Visser, 2018a), the presence of hypocapnia (Laffey & Kavanagh, 2002; Novak et al., 1998; Sato et al., 2012; Stewart et al., 2018a), endothelial dysfunction (Newton et al., 2012; Scherbakov et al., 2020), and the presence of antibodies against beta‐adrenergic receptors (Loebel et al., 2016; Tanaka et al., 2003; Yamamoto et al., 2012). As CBF is tightly coupled to the cerebral metabolic demands (Cipolla, 2010), a reduction in CBF may also be due to a temporarily reduced metabolic demand of the brain.

A limited number of studies in ME/CFS patients have investigated the effect of CO_2_ on CBF during orthostatic stress testing (Naschitz et al., 2006; Natelson et al., 2007, 2022; van Campen, Verheugt, et al., 2020). Most of the studies on orthostatic intolerance and hypocapnia have focused on postural orthostatic tachycardia syndrome (POTS) (Benarroch, 2012; Novak et al., 1998; Stewart et al., 2006, 2018b; Taneja et al., 2011; Tani et al., 2000), but others have found that CBF can also be reduced in the presence of hypocapnia but without POTS (Novak, 2018; Shin et al., 2016). Moreover, in the abovementioned ME/CFS studies, a cutoff value for CO_2_ was used to discriminate between patients with and without hypocapnia, while experimental data showed that there is a positive relation between the degree of CO_2_ reduction and the degree of CBF reduction (Willie et al., 2012).

Therefore, the aim of the study was to explore the relation between the degree of hypocapnia and the degree of CBF reduction in ME/CFS patients. For comparison, healthy controls were studied. As there are possible differences in CO_2_ reactivity between men and women (Deegan et al., 2011), and in light of the higher prevalence of ME/CFS in women, we elected to restrict the analysis to female patients and controls. Moreover, because of the hemodynamic differences in the tilt response of patients with a normal heart rate (HR) and blood pressure (BP) response and patients with POTS, we separately analyzed female patients with these two hemodynamic profiles.

## 2 MATERIALS AND METHODS

### 2.1 Eligible participants

We searched the database of the Stichting CardioZorg for all female ME/CFS patients who underwent tilt testing between October 2012 and July 2021 because of a clinical suspicion of OI, and in whom a complete set of data was available. We included female patients with ME/CFS who met both the criteria for CFS (Fukuda et al., 1994) and ME (Carruthers et al., 2011), taking the exclusion criteria into account. No alternative diagnosis was available to explain the symptomatology. Disease severity had been scored according to the ME criteria: mild: approximately 50% reduction in activity, moderate: mostly housebound, severe: mostly bedbound, and very severe: bedbound and dependent on help for physical functions (Carruthers et al., 2011). We have previously validated this severity classification (van Campen, Rowe, & Visser, 2020c). A completed set of data consisted of a tilt test with HR, BP, end‐tidal CO_2_ (P_ET_CO_2_), and CBF measurements. From the database, we selected patients with a normal HR and BP response as well as patients showing POTS (Fedorowski et al., 2009; Freeman et al., 2011; Sheldon et al., 2015; Shen et al., 2017). Patients with a BMI > 40 were excluded as hypoventilation may lead to higher end‐tidal CO_2_ (P_ET_CO_2_) levels (Nowbar et al., 2004; Reeves et al., 2003). We excluded patients unable to discontinue HR and BP‐lowering drugs, as well as patients using asthma/COPD medication with sympathomimetics (Jabre et al., 2009). Patients using HR or BP‐lowering drugs or the asthma/COPD medications who could tolerate discontinuing these were included if they had stopped the medications 1 week before the tilt test. Individuals being treated with selective serotonin reuptake inhibitors or serotonin‐norepinephrine reuptake inhibitors continued to take these medications. Patients using neuropathic pain medication (opioids, anti‐depressants, anti‐epileptics, and low‐dose naltrexone) were also allowed to continue the medication.

For comparison, data from 34 female healthy controls were included with all the required study parameters, all of whom had a normal HR and BP response during the tilt test. These controls were recruited from three sources: (a) announcements on ME/CFS patient advocacy websites, (b) posters in the medical clinic's office building, and (c) healthy acquaintances of the ME/CFS participants. None had a chronic illness, and none used chronic medication. Patients studied in the clinic for analysis of syncope, where no abnormalities were registered, were not considered to be healthy controls.

The study was carried out in accordance with the Declaration of Helsinki. The use of clinical data for descriptive studies (PT1450) and the use of healthy controls (P1411) were approved by the ethics committee of the Slotervaart Hospital, the Netherlands. All patients and controls gave informed consent.

### 2.2 Tilt table test

The tilt test was performed as described previously (van Campen et al., 2018b). Briefly, testing was conducted at least 3 h after a light meal. Participants were encouraged to ingest an ample amount of fluid on the day of the procedure, but not to drink fluids in the 2 h before the test. Participants were studied in a climate‐controlled room where the temperatures range from 22 to 24°C. They were studied in the supine position for 15 min, and for a maximum of 30 min in the upright position (70 degrees). The test was completed after 30 min or ended earlier at the request of the participant because of severe complaints, or when they developed syncope or pre‐syncope.

HR, systolic, and diastolic BP (SBP and DBP) were continuously recorded by finger plethysmography (Eeftinck Schattenkerk et al., 2009; Martina et al., 2012). HR and BP data were extracted from the finger plethysmography device and imported into an Excel spreadsheet. Supine HR and BP data were calculated from the last‐minute data before tilting. Upright HR and BP data were calculated from the last‐minute data of the upright position and denoted to as end‐tilt. For P_ET_CO_2_ measurements, the Nonin Lifesense II (Nonin Medical Inc.) was used, connected to nasal prongs.

### 2.3 Cerebral blood flow measurements

Measurements were performed as described previously (van Campen et al., 2018b). Internal carotid artery and vertebral artery Doppler flow velocity frames were acquired by one operator (FCV), using a Vivid‐I system (GE Healthcare, Hoevelaken, the Netherlands) equipped with a 6–13 MHz linear transducer. High‐resolution B mode images, color Doppler images, and the Doppler velocity spectra (pulsed wave mode) were recorded in one frame. At least two consecutive series of six frames per artery were recorded. The recording time intervals of the first and last imaged artery were noted, and these times were corrected to the times of a radio clock, setting the start of tilt at 0 min. HR and BP of the echo recording time intervals were averaged. Images were acquired supine and during standing. Image acquisition for all four vessels lasted approximately 3 (1) min.

Blood flow of the internal carotid and vertebral arteries was calculated offline by an investigator (CMCvC) who was unaware of the patient or control status and unaware of the hemodynamic outcome of the head‐up tilt test. The vessel surface area was calculated from the mean diameter as proposed by Sato et al. (2011): mean diameter = (peak systolic diameter*1/3) + (end‐diastolic diameter*2/3) (Sato et al., 2011). Blood flow in each vessel was calculated from the mean blood flow velocities x the vessel surface area and expressed in mL/min. Flow in the individual arteries was calculated in 3–6 cardiac cycles, and data were averaged. Total CBF was calculated by adding the flow of the four arteries. We previously demonstrated that this methodology had good intra‐ and inter‐observer variability (van Campen et al., 2018b).

### 2.4 Statistical analysis

Data were analyzed using the statistical package of SPSS, version 21 (IBM). All continuous data were tested for normal distribution using QQ plots in combination with histograms and presented as mean (SD) or as median (IQR), where appropriate. Nominal data (disease severity) were compared using the Chi‐square test. For the comparison of single data of two independent groups the unpaired *t*‐test or the Mann–Whitney *U* test was used, where appropriate. For comparison of two data within a group, the paired *t*‐test or the Wilcoxon signed‐rank test was used where appropriate. For multiple comparisons, the ordinary one‐way analysis of variance (ANOVA) or Kruskal–Wallis H test was used. Where significant, results were explored further using the post hoc Tukey's test or Dunn's test.

A multiple regression was performed on the supine data taking independence, homoscedasticity, and normal distribution of residuals, multicollinearity, outliers, leverage, and influential points into account (Statistics, 2015). For the prediction of the supine CBF in patients, P_ET_CO_2_, age, disease severity with dummy variables for moderate and severe disease, disease duration, the type of hemodynamic tilt test result (normal HR and BP versus POTS), supine HR, and supine MAP were analyzed. The same analysis was performed for the prediction of end‐tilt CBF, using end‐tilt P_ET_CO_2_, HR, and MAP, instead of supine data. For the prediction of the %CBF decrease, delta P_ET_CO_2_, delta HR, and delta MAP were used. A separate analysis was performed for the combined patient, and healthy controls. P_ET_CO_2_, age, the type of hemodynamic tilt test result (normal HR and BP versus POTS), HR, MAP, and patients versus healthy controls were analyzed.

Linear regression lines were constructed using GraphPad Prism version 6.05 (GraphPad software). Using the same software, regression lines were compared to determine whether slopes were significantly different. Due to the large number of comparisons, a *p*‐value of <0.01 was considered significant.

## 3 RESULTS

**FIGURE 1:**
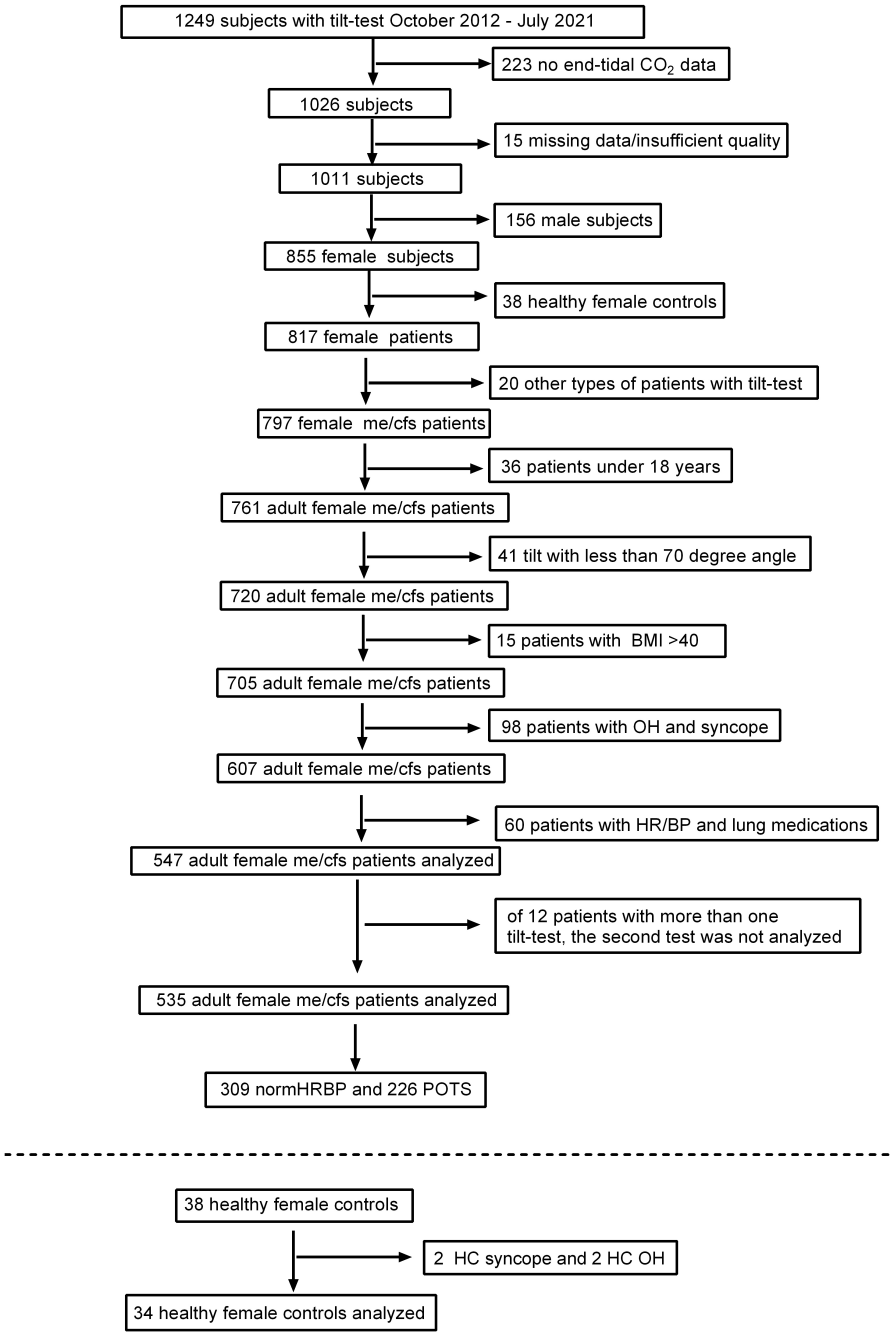
Patients/healthy controls flow of the inclusion and exclusion criteria. BMI: body mass index; normHRBP, normal heart rate/blood pressure during tilt test; ME/CFS, myalgic encephalomyelitis/chronic fatigue syndrome; OH, orthostatic hypotension; POTS, postural orthostatic tachycardia syndrome during tilt test.

Figure 1 shows the patient flow. A total of 535 female ME/CFS patients and 34 female healthy controls were included in the study.

Table 1 shows the baseline characteristics of ME/CFS patients with a normal HR and BP response during the tilt test, of patients with POTS and of the healthy controls. Patients with a normal HR and BP response were significantly older (*p* < 0.0001) and had a higher BMI (*p* < 0.0001) than the other two groups. Disease duration was significantly shorter in the POTS patients compared with the patients with a normal HR and BP response (*p* < 0.0001), but disease severity was significantly worse in POTS patients (*p* = 0.004). Very severely diseased patients were not included in the study as they were not able to tolerate a tilt test.

**TABLE 1:** Demographic data of the study population.

	ME/CFS norm HRBP (*n* = 309) group 1	ME/CFS POTS (*n* = 226) group 2	Healthy controls norm HRBP (*n* = 34) group 3	*p*‐value one‐way ANOVA with post hoc Tukey's test or Kruskal–Wallis H‐test with post hoc Dunn's test or chi‐squared or Mann–Whitney test
Age (years)	42 (11)	34 (9)	34 (14)	*F*(2, 566) = 42.73, *p* < 0.0001; 1 vs. 2: *p* < 0.0001; 1 vs. 3: *p* < 0.0001
Height (cm)	169 (7)	172 (7)	171 (5)	*F*(2, 566) = 10.35, *p* < 0.0001; 1 vs. 2: *p* < 0.0001
Weight (kg)	69 (60–80)	66 (59–75)	64 (62–78)	X^2^ (2) = 6.58, *p* = 0.04
BSA (duBois m^2^)	1.79 (1.69–1.93)	1.77 (1.68–1.90)	1.77 (1.70–1.90)	X^2^ (2) = 1.45, *p* = 0.48
BMI (kg/m^2^)	24 (21–28)	22 (20–25)	22 (21–26)	X^2^ (2) = 18.32, *p* = 0.0001; 1 vs. 2: *p* < 0.0001
Disease Dur (yrs)	12 (7–20.5)	8.5 (4–14.25)	na	*p* < 0.0001^†^
Severity* (1,2,3%)	91/173/45 29/56/15%	54/113/59 24/50/26%	na	*p* = 0.004^#^

Abbreviations: BMI, body mass index; BSA, body surface area; formula of Dubois; Dur, duration; HC, healthy controls; na, not applicable; Norm HRBP, normal heart rate and blood pressure response during tilt test; POTS, postural orthostatic tachycardia syndrome during tilt test.

^#^ Chi‐square test.

^†^ Mann–Whitney test.

* ME severity criteria (Carruthers et al., 2011).

Table 2 shows the hemodynamic results of the tilt test. HR in the supine position was higher in those with POTS than in the two other groups (*p* < 0.0001 and *p* < 0.0001). As expected, HR at end‐tilt in POTS patients was higher than in the other two groups (both *p* < 0.0001). In patients with a normal HR and BP response, end‐tilt SBP was significantly higher than in POTS patients (*p* < 0.0001). MAP was significantly higher at end‐tilt in patients with a normal HR and BP response compared to the healthy controls (*p* = 0.0004). P_ET_CO_2_ in the supine position did not differ between the three groups but a significant difference was found at end‐tilt with the lowest P_ET_CO_2_ and largest P_ET_CO_2_ decrease during the tilt test in POTS patients compared with the other two groups; the highest P_ET_CO_2_ values and the smallest decrease during the tilt were observed in the healthy controls (all *p* < 0.0001). CBF in the supine position was similar in the three groups, but in both patient groups, the end‐tilt CBF was significantly lower than in healthy controls (both *p* < 0.0001). Moreover, the percent change in CBF at end‐tilt versus the supine tilt was highest for the POTS patients and lowest for the healthy controls (all *p* < 0.0001).

**TABLE 2:** Tilt test hemodynamic data of healthy controls and ME/CFS patients.

	1 ME/CFS norm HRBP (*n* = 309)	2 ME/CFS POTS (*n* = 226)	3 HC norm BPHR (*n* = 34)	*p*‐value one‐way ANOVA with Tukey's post hoc test
HR supine (bpm)	73 (11)	78 (12)	70 (11)	*F*(2, 566) = 16.08, *p* < 0.0001; 1 vs. 2: *p* < 0.0001, 2 vs. 3: *p* = 0.0004
HR end‐tilt (bpm)	88 (12)	115 (17)	84 (14)	*F*(2, 566) = 238.1, *p* < 0.0001; 1 vs. 2: *p* < 0.0001; 2 vs. 3: *p* < 0.0001
SBP supine (mmHg)	136 (18)	132 (16)	133 (16)	*F*(2, 566) = 3.45, *p* = 0.03
SBP end‐tilt (mmHg)	134 (19)	126 (21)	125 (14)	*F*(2, 566) = 11.74, *p* < 0.0001; 1 vs. 2: *p* < 0.0001
DBP supine (mmHg)	81 (12)	80 (10)	78 (7)	*F*(2, 566) = 0.72, *p* = 0.49
DBP end‐tilt (mmHg)	88 (14)	87 (15)	81 (9)	*F*(2, 566) = 3.84; *p* = 0.02
MAP supine (mmHg)	103 (13)	101 (12)	97 (9)	*F*(2, 566) = 3.66, *p* = 0.03
MAP end‐tilt (mmHg)	106 (15)	104 (17)	95 (10)	*F*(2, 566) = 7.84, *p* = 0.0004; 1 vs. 3: *p* = 0.0004
P_ET_CO_2_ supine (mmHg)	37 (3)	36 (3)	37 (3)	*F*(2, 566) = 3.53, *p* = 0.03
P_ET_CO_2_ end‐tilt (mmHg)	30 (5)	26 (5)	36 (3)	*F*(2, 566) = 61.80, *p* < 0.0001; 1 vs. 2: *p* < 0.0001; 1 vs. 3: *p* < 0.0001; 2 vs. 3: *p* < 0.0001
Delta CO_2_ end‐tilt min supine (mmHg)	−7 (4)	−10 (4)	−2 (1)	*F*(2, 566) = 65.44, *p* < 0.0001; 1 vs. 2: *p* < 0.0001; 1 vs. 3: *p* < 0.0001; 2 vs. 3: *p* < 0.0001
CBF supine (mL/min)	610 (101)	620 (98)	621 (82)	*F*(2, 566) = 0.68, *p* = 0.51
CBF end‐tilt (mL/min)	442 (84)	429 (74)	577 (79)	*F*(2, 566) = 51.01, *p* < 0.0001; 1 vs. 3: *p* < 0.0001; 2 vs. 3: *p* < 0.0001
%CBF end‐tilt—supine (mL/min)	−28 (7)	−31 (6)	−7 (2)	*F*(2, 566) = 197.4, *p* < 0.0001; 1 vs. 2: *p* < 0.0001; 1 vs. 3: *p* < 0.0001; 2 vs. 3: *p* < 0.0001

Abbreviations: CBF, cerebral blood flow; DBP, diastolic blood pressure; HC, healthy controls; HR, heart rate; MAP, mean arterial pressure; Norm HRBP, normal heart rate and blood pressure response during tilt test; P_ET_CO_2_; end‐tidal carbon dioxide; POTS, postural orthostatic tachycardia syndrome during tilt test; SBP, systolic blood pressure.

**FIGURE 2:**
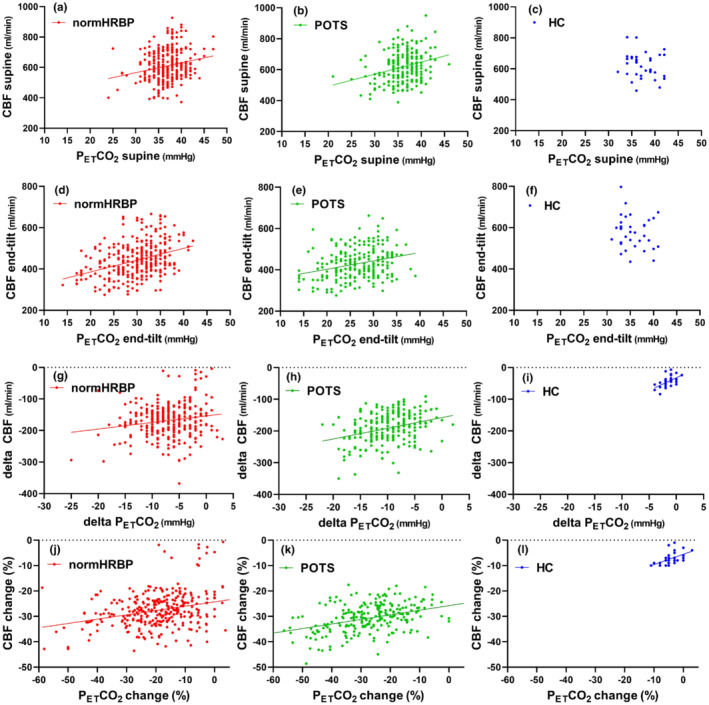
(a–l) Relation between P_ET_CO_2_ and CBF in ME/CFS patients with a normal heart rate and blood pressure response and with postural orthostatic tachycardia syndrome. CBF, cerebral blood flow; normHRBP, normal heart rate/blood pressure during tilt test; P_ET_CO_2_, end‐tidal carbon dioxide; POTS, postural orthostatic tachycardia syndrome during tilt test.

Figure 2 shows the comparison of the relation between the P_ET_CO_2_ and CBF in patients with a normal HR and BP response versus patients with POTS. Both in the supine position and at end‐tilt the relations were highly significant (all four: *p* < 0.0001). As slopes and Y‐axis intercepts were not different between the two patient groups in the supine position and at end‐tilt, the data of patients were grouped together for the supine position and for the end‐tilt data.

**FIGURE 3:**
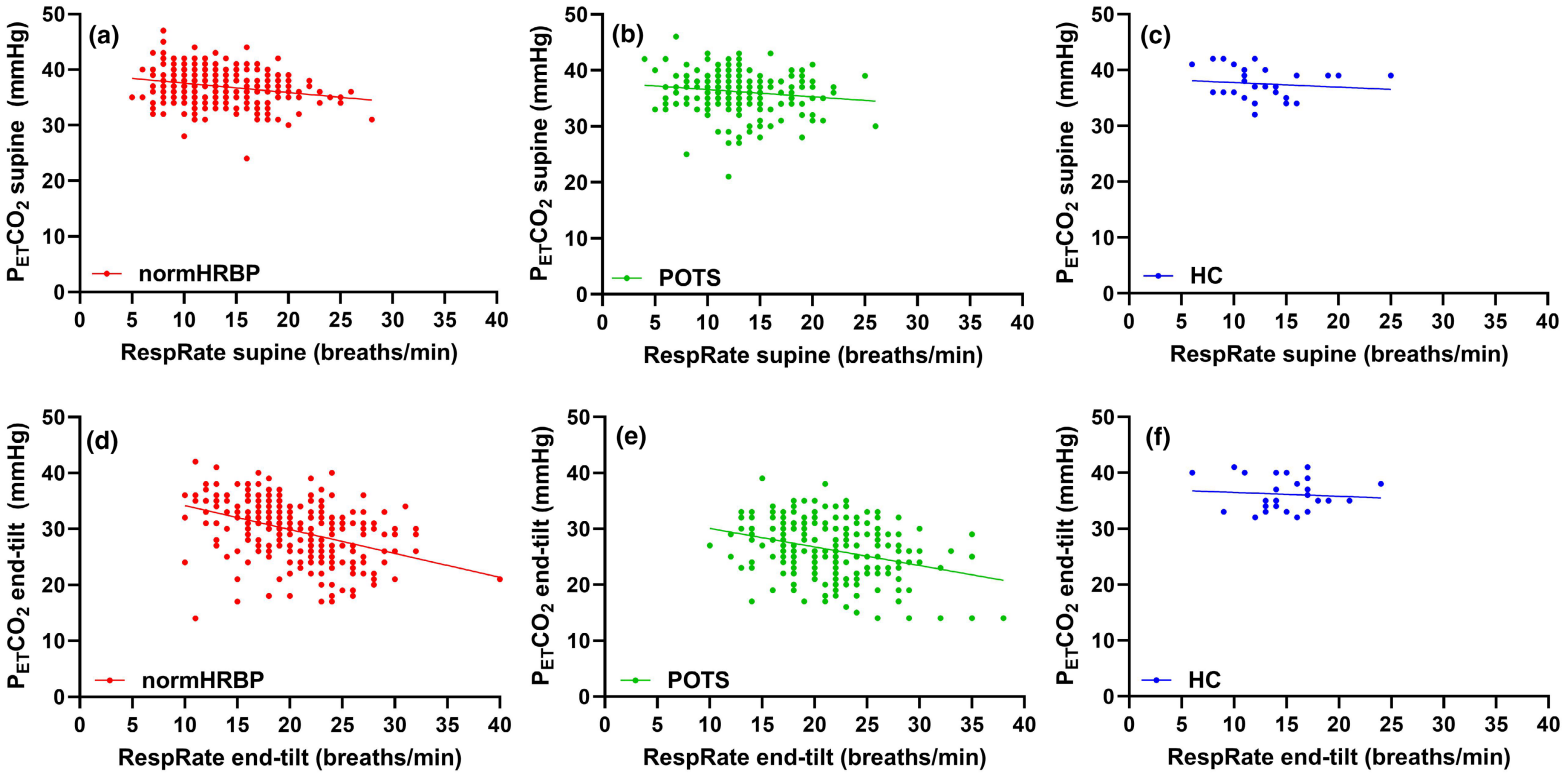
(a–f) Relation between P_ET_CO_2_ and CBF in all ME/CFS patients and healthy controls. CBF, cerebral blood flow; P_ET_CO_2_, end‐tidal carbon dioxide.

Figure 3 shows the relation between the P_ET_CO_2_ and CBF in the supine position, at end‐tilt, and the CO_2_ reactivity, expressed as the relation between delta P_ET_CO_2_ and the %CBF decrease (Willie et al., 2012) in all ME/CFS patients and healthy controls. In patients, the relation between P_ET_CO_2_ and CBF was highly significant in the supine position and at end‐tilt (both *p* < 0.0001). In healthy controls, the relations between the P_ET_CO_2_ and CBF supine and end‐tilt were not significantly different from zero. The CO_2_ reactivity data were significant in both patients and healthy controls (*p* < 0.0001 and *p* = 0.0012, respectively). The slopes of the relation between delta P_ET_CO_2_ and %CBF decrease were not significantly different. As shown in Figure 3, there were 12 patients with a limited %CBF and delta P_ET_CO_2_ change. When considering these 12 patients as outliers, the slope of the %CBF–P_ET_CO_2_ relationship in the remaining patients changed from 0.5333 to 0.3966. Despite this lower slope, the slopes in patients and healthy controls remained not significantly different.

The multiple regression analysis to predict CBF is shown in Table 3. In ME/CFS patients only, supine P_ET_CO_2_ and age contributed significantly to the prediction of supine CBF (*P*_model_ < 0.0001). End‐tilt CBF was only predicted by end‐tilt P_ET_CO_2_ (*P*_model_ < 0.0001). The %CBF decrease was predicted by delta P_ET_CO_2_ and age (*P*_model_ < 0.0001). Other variables did not significantly contribute. In the combined analysis of patients and healthy controls supine, P_ET_CO_2_ and age contributed significantly to the prediction of supine CBF (*P*_model_ < 0.0001). End‐tilt CBF was predicted by end‐tilt P_ET_CO_2_ and by the patient versus healthy controls division (*P*_model_ < 0.0001). The %CBF decrease was predicted by delta P_ET_CO_2_ and by the patient versus healthy controls division (*P*_model_ < 0.0001). Other variables did not significantly contribute.

**TABLE 3:** Multiple regression analysis to predict CBF from other variables.

Variable	CBF supine	CBF end‐tilt	%CBF decrease	CBF supine	CBF end‐tilt	%CBF decrease
	Patients (*n* = 535)			Patients and healthy controls (*n* = 569)		
P_ET_CO_2_ ^a^	7.222	4.943	0.432	6.656	4.897	0.487
Age	−1.186	ns	0.083	−1.146	ns	ns
HR^a^	ns	ns	ns	ns	ns	ns
MAP^a^	ns	ns	ns	ns	ns	ns
Pat‐HC	–	–	–	ns	−104.273	−18.188
Disease duration	ns	ns	ns	–	–	–
Moderate disease	ns	ns	−2.485	–	–	–
Severe disease	ns	ns	−3.077	–	–	–
normBPHR‐POTS	ns	ns	ns	ns	ns	ns
Constant	332.47	297.65	−25.445	349.30	391.76	−8.369
P of model	<0.0001	<0.0001	<0.0001	<0.0001	<0.0001	<0.0001
Adj *R* ^2^ of model	0.07	0.13	0.19	0.07	0.24	0.47

Abbreviations: Adj, adjusted CBF, cerebral blood flow; HC, healthy controls; HR, heart rate in bpm; MAP, mean arterial pressure; moderate and severe disease: ME disease severity criteria (Carruthers et al., 2011); normHRBP, normal heart rate and blood pressure during tilt test; P, significance of the model; Pat, ME/CFS patients; P_ET_CO_2_, end‐tidal carbon dioxide pressure in mmHg; POTS, postural orthostatic tachycardia syndrome during tilt test; *R*, correlation coefficient of the model.

^a^ P_ET_CO_2_, heart rate and mean arterial pressure data of supine, end‐tilt, and the differences, were taken for the supine, end‐tilt, and CO_2_ reactivity analysis, respectively.

Based on the mean and SD of supine P_ET_CO_2_ in healthy controls of 37.18 ± 2.71 mmHg, we defined the lower limit of a normal supine P_ET_CO_2_ of 32 mmHg. Using this cutoff value, 34/535 (6%) patients had a supine P_ET_CO_2_ below 32 mmHg. Similarly, based on the mean and SD of end‐tilt P_ET_CO_2_ in healthy controls of 35.74 ± 2.78 mmHg, we defined the lower limit of a normal end‐tilt P_ET_CO_2_ as 30 mmHg. Using this cutoff value, 283/535 (53%) patients had an end‐tilt P_ET_CO_2_ below 30 mmHg. Based on the mean and SD of delta P_ET_CO_2_ in healthy controls of −2 ± 1 mmHg, we defined the lower limit of normal of −4 mmHg. Using this cutoff value, 429/535 (80%) patients had a delta P_ET_CO_2_ below −4 mmHg.

## 4 DISCUSSION

It has been demonstrated with a variety of techniques including PET, SPECT, MRI, and TCD (Juttukonda & Donahue, 2019) that the cerebral vasculature is susceptible to changes in CO_2_ concentrations, where an increase in *p*CO_2_ leads to arterial vasodilation and an increase in CBF, and a decrease in *p*CO_2_ to vasoconstriction and CBF reduction (Kety & Schmidt, 1946, 1948). This phenomenon has been named cerebrovascular CO_2_ reactivity and has been studied in a variety of cerebral diseases (Hoiland et al., 2019; Juttukonda & Donahue, 2019). In ME/CFS patients, an abnormal decrease in CBF has been demonstrated during a variety of orthostatic challenges (Stewart et al., 2012; van Campen, Rowe, & Visser, 2020a, 2020b; van Campen, Verheugt, et al., 2020). One of the proposed mechanisms for the abnormal CBF decrease is hypocapnia, where a *p*CO_2_ reduction leads to vasoconstriction of supplying cerebral arteries (Laffey & Kavanagh, 2002; Novak et al., 1998; Sato et al., 2012; Stewart et al., 2018b). The hypocapnia in ME/CFS patients is hypothesized to be related to baroreflex unloading during orthostatic stress, which stimulates peripheral oxygen‐dependent chemoreflexes causing hyperventilation (Medow et al., 2014). Most studies in ME/CFS patients were conducted in a limited number of patients, with sample sizes ranging from 10 to 90, (median *N* = 25) (Medow et al., 2014; Natelson et al., 2007, 2022; Novak et al., 1998; Sato et al., 2012; Stewart et al., 2018a, 2018b; Taneja et al., 2011; Tani et al., 2000). Therefore, we assessed the relation between P_ET_CO_2_ and CBF in a large number of female ME/CFS patients, both in the supine position and during orthostatic stress, as well as the relation between the changes in P_ET_CO_2_ and changes in CBF and compared the data with those of healthy controls. As there are possible differences in CO_2_ reactivity between men and women (Deegan et al., 2011), we elected to restrict the analysis to women, who have a higher prevalence of ME/CFS. Moreover, most prior studies were performed in patients with POTS. We studied a more representative population of ME/CFS patients, including those with a normal HR and BP response.

Even in the supine position, we observed a positive and linear relation between the P_ET_CO_2_ and CBF in the ME/CFS patient groups with a normal HR and BP response and with POTS (Figure 2), without differences between the two patient groups and without difference in supine CBF values compared with the healthy controls. In general, CBF is not only dependent on P_ET_CO_2_ levels but also dependent on age, P_ET_CO_2_, blood pressure, neuronal activity, gender, exercise, and sleep (Hoiland et al., 2019). In ME/CFS patients, the presence of endothelial dysfunction (Newton et al., 2012; Scherbakov et al., 2020; Sørland et al., 2021), auto‐antibodies (Meyer & Heidecke, 2018; Tanaka et al., 2003; Wirth et al., 2021), and a reduced circulating blood volume (Farquhar et al., 2002; Hurwitz et al., 2010; Streeten et al., 2000; van Campen, Rowe, & Visser, 2018; van Campen & Visser, 2018b) need to be considered and these complex relationships deserve future research. Nevertheless, the supine CBF versus P_ET_CO_2_ relation fell into the normal range of healthy controls in 94% of patients. This suggests that the abovementioned abnormalities in ME/CFS patients were of limited significance in the supine position. The multiple regression analysis also showed that supine CBF was inversely related to age, consistent with the findings of previous studies (for a review, see Tarumi & Zhang, 2018).

Similarly, the same observation of the positive relation between P_ET_CO_2_ and CBF could be found for the end‐tilt data. Based on the end‐tilt data of P_ET_CO_2_, where end‐tilt P_ET_CO_2_ values were lower in patients with POTS, we expected a difference in the relation between P_ET_CO_2_ and CBF in patients with a normal HR and BP response versus patients with POTS. However, the slopes of the regression lines were not significantly different, as well as the Y‐axis intercept, both in the supine position and at end‐tilt (Figure 2). POTS is a complex disease and pathophysiological mechanisms involve hypovolemia, neuropathy, and hyperadrenergic states, as well as involvement of the immune system, mast cell disorders, physical deconditioning, norepinephrine transporter deficiency, and impaired cerebral autoregulation (Lloyd & Raj, 2021). However, many of the mechanisms may also be involved in the ME/CFS patients with a normal HR and BP response during the tilt. Moreover, POTS has been shown to have a diurnal variation with more patients showing POTS in the morning compared with the evening (Brewster et al., 2012). This suggests that patients with POTS in the morning may shift to a normal HR and BP response in the evening. Our data showing that the response of CBF to P_ET_CO_2_ is not different between patients with a normal HR and BP response during the tilt and patients with POTS suggest a similar response of CBF to P_ET_CO_2_ in both groups. This is also evident from the multiple regression analysis. On the contrary, one mechanism changing the CBF versus P_ET_CO_2_ relationship in one group (e.g., POTS patients) may be compensated by another mechanism in the other group (e.g., in patients with a normal HR and BP response). This needs to be studied further. From Figure 3 and the multiple regression, it is obvious that patients have a larger decrease in CBF than healthy controls. This larger decrease is also related to the cardiac output, as we (van Campen & Visser, 2018a) and others (Timmers et al., 2002) have previously shown that cardiac/stroke volume index decreases to a larger extent in ME/CFS patients compared to healthy controls. Although the cardiac output is the major determinant, the present multiple regression shows that also P_ET_CO_2_ contributes to the CBF reduction. Both mechanisms are amendable as we have recently shown that compression stockings improve symptoms and cardiac index during tilt testing (van Campen et al., 2018a, 2021) and as Stewart et al. (2018b) showed that addition of exogenous CO_2_ improved symptoms and cardiac output in POTS patients during tilt testing.

Figure 3 also shows that CO_2_ reactivity, defined by the slope of the change in P_ET_CO_2_ versus the %CBF decrease during the tilt, was significant in patients. We further explored the dependence of the %CBF change on other variables than delta P_ET_CO_2_. The %CBF change was also dependent on age and the ME/CFS severity. Moderate and severe ME/CFS patients had a larger %CBF decrease during the tilt. Our data contrast the observation by Natelson et al. (2007), that disease severity in 62 patients was not related to the presence or absence of hypocapnia; however, they used a fixed cutoff value of 30 mmHg for the P_ET_CO_2_ and did not measure cerebral blood flow directly. These CO_2_ reactivity findings in ME/CFS patients have not been published before in as large a sample of patients. Comparing the slope of the %CBF reduction versus the delta P_ET_CO_2_ in patients and healthy controls, Figure 3 shows that the slopes were not significantly different. Our data therefore suggest that CO2 reactivity is unaltered in ME/CFS patients, which contrasts the findings in other patient populations, such as diabetes, dementia, and stroke (Hoiland et al., 2019). However, the range of P_ET_CO_2_ changes in healthy controls during orthostatic stress were limited and a larger range of P_ET_CO_2_ reductions, for example, by forced hyperventilation may enlarge the range of changes in P_ET_CO_2_, thereby increasing the accuracy of the comparison between patients and healthy controls.

Many studies have shown that excitability of neurons is pH dependent: lowering the pH results in reduced excitability and increased pH increases neuronal excitability (Sinning & Hübner, 2013; Xu et al., 2010). We found that end‐tilt P_ET_CO_2_ was reduced in 53% of patients, being lower than the lower limit of normal (30 mmHg), and in 80% of patients, a delta P_ET_CO_2_ below the lower limit of −4 mmHg was found. The multiple regression analysis showed that moderate and severe ME/CFS patients had a larger P_ET_CO_2_ decrease than patients with a mild disease. The data suggest that disease severity (and maybe disease progression) is negatively influenced by the degree of hypocapnia. To answer this hypothesis, a follow‐up study is needed. Furthermore, Fahti et al. (2011) showed in a model of human cerebral microvascular endothelium and astrocytes that hypocapnia induced an decrease in NO levels in endothelial cells of 30%, whereas NO levels in astrocytes were unchanged (Fathi et al. 2011). The NO decrease in endothelial cells was prevented by L‐arginine (an NO donor), linking the role of NO to hypocapnia‐induced vasoconstriction. NO plays an important role in endothelium‐derived vasodilation (Tousoulis et al., 2012). As endothelial dysfunction has been demonstrated in ME/CFS patients (Newton et al., 2012; Scherbakov et al., 2020; Sørland et al., 2021), it is tempting to relate the lowered P_ET_CO_2_ values in ME/CFS patients with endothelial dysfunction. This also needs to be established in the future.

An interesting subgroup of patients with a normal HR and BP response are those who show a limited change in %CBF reduction, and whose values of %CBF change and delta P_ET_CO_2_ values are in the range of the healthy controls (Figure 3b). These patients may be a subset of ME/CFS patients, but larger numbers of these patients are needed to define their characteristics and the differences compared with the other ME/CFS patients. A subgroup of ME/CFS patients without orthostatic hypocapnia was also described by Natelson et al. (2007).

Although we found significant relations between the P_ET_CO_2_ and CBF, the contribution of the P_ET_CO_2_, age, and disease severity to the CBF changes is limited. This can be inferred from the low adjusted *R* ^2^ values in the regression analysis, being 0.19 in the %CBF versus the delta P_ET_CO_2_ analysis of the patients. This indicates that only 19% of the changes in CBF can be explained by the studied variables (Miles, 2014). Other authors have suggested that hypocapnia is the driving force in CBF reduction (Natelson et al., 2007, 2022; Stewart et al., 2018a, 2018b). In the current study, we showed that the hypocapnia only plays a minor role in explaining the CBF reduction. The reduction in venous return to the heart and subsequently the reduction in cardiac output are likely to be the most important factors (van Campen & Visser, 2018a) in ME/CFS patients. This needs to be evaluated in greater detail in future studies.

Methodological considerations: we used the orthostatic stress to provoke P_ET_CO_2_ changes and used the slope of the relation between P_ET_CO_2_ versus CBF of the collective group of patients and healthy controls to assess CO_2_ reactivity. Most studies used a CO_2_ intervention with paired testing of pre and post the CO_2_ intervention: see review Hoiland et al. (2019). Our data suggest that the analysis of the collective group of patient is valid and may be used in future studies in other patient populations. Furthermore, a direct comparison between the orthostatic P_ET_CO_2_ group changes and CO_2_ intervention is needed.

### 4.1 Limitations

We cannot exclude the possibility that referral bias may have created a study sample that differs from the general population of those with ME/CFS. However, we studied patients of all disease severities except for the very severe, as they would have an increase in functional impairment due to the tilt testing superimposed on their very serious condition. Our focus was on correlating CBF supine and during head‐up tilt testing with end‐tidal CO_2_; investigations of regional CBF and arterial *p*CO_2_ are beyond the scope of this study and are important aspects to be studied in future. Finally, in the present study we investigated only women as they may have a different CO_2_ reactivity compared to men (Deegan et al., 2011). The CO_2_ reactivity in male ME/CFS patients deserves further studies. Also, the CO_2_ reactivity needs to be determined in ME/CFS patients with orthostatic hypotension and/or syncope during tilt testing.

## 5 CONCLUSIONS

In female ME/CFS patients, CO_2_ reactivity, as measured during tilt testing, is similar to that of healthy controls and is independent of the type of hemodynamic abnormality of the tilt test (normal HR and BP response/POTS), but is dependent on age and the severity of the disease. Our data also show that the influence of CO_2_ changes on CBF changes is limited in these female ME/CFS patients.

## AUTHOR CONTRIBUTIONS

CMCVC, PCR, and FCV conceived the study. CMCVC and FCV collected the data. CMCVC performed the primary data analysis. FCV and PCR performed secondary data analyses. All authors were involved in the drafting and review of the manuscript.

## FUNDING INFORMATION

This study was performed without grant funding.

## CONFLICT OF INTEREST STATEMENT

The authors declare that the research was conducted in the absence of any commercial or financial relationships that could be construed as a potential conflict of interest.
